# The Role of Body in Brain Plasticity

**DOI:** 10.3390/brainsci12020277

**Published:** 2022-02-17

**Authors:** Mariella Pazzaglia

**Affiliations:** 1 Dipartimento di Psicologia, Università di Roma “Sapienza”, Via dei Marsi 78, 00185 Roma, Italy; mariella.pazzaglia@uniroma1.it; 2 Body and Action Lab, IRCCS Fondazione Santa Lucia, Via Ardeatina 306, 00179 Rome, Italy

Our bodily experience arises primarily from the integration of sensory, interoceptive, and motor signals and is mapped directly into the sensorimotor cortices [1]. This view predicts major changes in body representation with changes in sensorimotor experience generated directly by current sensory input and motor control. Deafferentation/De-efferentation events, such as spinal cord trauma, brachial plexus injury, or amputation, often have dramatic effects on sensorimotor cortices by silencing all sensory and proprioceptive signals that flow into the primary somatosensory and primary motor cortex and activate motor control over the limbs. To compensate for sensorimotor loss, central nervous system remodeling may occur in the brain’s white and gray matter by aberrant signaling [2,3,4]; progressive atrophic, microstructural, and biochemical changes [5,6,7,8]; an imbalance in excitation or inhibition [9], or a spatial shift in functional sensorimotor representation [10]. Cortical areas become muted when they fail to respond to stimulation, as occurs following brachial plexus and spinal cord damage, or they become maladaptive, as happens with the development of neuropathic pain and phantom sensations such as those associated with amputations [1,11,12,13]. However, despite this reorganization, the missing limbs remain strongly represented [14,15]. The brain might maintain a relatively persistent offline representation of its own body to reflect a more permanent and diachronic aspect of the body, that is, a code in the sensorimotor structure [16]. Moreover, experimental studies show that both somatosensory stimulation and local anesthesia produce changes in body perception such as phantom limbs, but these changes do not outlast changes in sensorimotor traffic [17]. These views receive some empirical support, suggesting that body representation is indeed a synthesis of multiple information sources. Although the synthesis of the online and offline body representations are unclear [18], these two representations may be linked to test the sense of embodiment (the feeling of one’s own body) and sense of agency (SoA; the sense that one’s body controls one’s actions) and abnormalities in their relationship may occur in various clinical disorders.

The present collection of articles emphasizes the relevance of bodily and action systems for higher-order cognitive processes and self-awareness [19]. It assimilates interdisciplinary findings from neuropsychology, neurology, and cognitive psychology, and underlines that when viewed from an action, the body systems emerge in cognitive operations such as perception, reasoning, and thinking.

The bodily instantiation of cognitive operations is referred to as “embodiment” and is equated with actions such as symbolic processing, emotion, language, and attention [19]. Nevertheless, limited evidence exists for embodied cognition approaches, but in this Special Issue, two important experimental contributions to memory and creativity appear. On memory, Piccardi et al. [20] found that participants exposed to both negative and positive rather than neutral landmarks more quickly learned the situational path connecting the landmarks. The results showed that the embodied landmarks improved path learning.

Palmiero et al. [21] emphasized that visual creativity related to object originality can be embodied, to some extent, as perceptuo-motor codes, suggesting that simulations and other forms of knowledge representation permeate grounded cognition. Many studies in this Special Issue instead address embodiment in the context of the bodily awareness as the start for the sense of ownership (the sense that one’s body is one’s own) and the sense of *agency*. Regarding the role of body feeling in clinical practice, and especially in neurorehabilitation, one study highlighted the high relevance of bodily processes considering the clinical contributions of different pathologies (tumor, brain, and spinal cord lesions). The authors introduced convergent studies on neural damage to the fronto-parietal-insular network that disrupts body sensation, which can lead to a sense of disembodiment or a sense of disownership [22]. This perspective suggests that the need to restore a normal sense of body ownership after clinical disturbances of the physical body may facilitate the selection of appropriate and more effective rehabilitation treatments.

Bodily awareness can be quite easily manipulated in healthy participants by the rubber hand illusion [23], and such paradigms could provide an interesting tool for clinical conditions involving physical loss of the body limb [12,24]. 

Sensorimotor representations are also essential for building and maintaining corporeal awareness. Indeed, not only does the mere motor imagination (MI) of an action enhance the motor representation of the imagined action [25], but the mere motor experience of a particular action can also enhance its representational organization [26].

Muscle-specific modulation of spinal reflexes in lower limb muscles during action observation, with and without motor imagination of walking, suggests that the muscle-specific facilitation of spinal reflexes is related to the strength of connectivity between the sensorimotor area and the muscle during the actual movement. 

Although research originally focused on the relationship between performed and observed actions, more recent studies highlight the importance of multimodal experiences modulated by auditory [27], somatic [24,28,29], and even olfactory inputs [30]. The study of Grant et al. [31] suggest that audiohaptic stimuli enhance motor performance and subjective ratings of realness in a drilling simulation, providing further evidence that action is inherently linked to the internal reproduction of perceptuo-motor states called ‘simulation,’ a process that supposedly enables the interindividual sharing of multisensory experiences.

This primary adaptive mode of perceptuo-motor control also requires an additional representational transformation to a body-centered, one-person perspective. The representational transformation is thought to embody imitation, which allows one to reproduce the actions of another person from a different perspective to achieve a goal. In an interesting study [32] on motor program transformation of throwing darts from the third to first person perspective, visuomotor transformation changes dynamically depending on the subject’s localization. This inherent functional and anatomical bidirectional connection between sensory and motor action may provide a deceptively simple mechanism for SoA. 

SoA may also be enhanced by detecting errors in one’s own actions with changes in the environment. In a study presented in this Special Issue [33], participants performed an implicit regularity perceptuo-motor task and an intentional binding task (a method that can quantitatively measure SoA) simultaneously. SoA was gradually enhanced in the group noticing the regularity of a perceptuo-motor task compared to that of the non-noticing group. The results suggest that the experience of noticing may enhance SoA during perceptuo-motor tasks, a crucial ability for more flexible behavior. These studies supporting the internal reproduction of perceptuo-motor states mapped onto modal sensorimotor cortices illustrate the extent that simulation and other forms of knowledge representation permeate grounded cognition. Multisensory training may be beneficial in preventing maladaptive brain reorganization and may yield useful information, leading to appropriate clinical treatment when altered sensory information is present. These findings could be relevant in cases in which the sense of agency changes according to the sensorimotor deficit severity and paretic upper limb activity [34] or, for example, in apraxia [35]. These stimulating results enhance our knowledge and interest for further basic and clinical investigations on the role of body and action in clinical and rehabilitation [26,36,37,38].

Taken together, the papers in this Special Issue have covered and expanded the surprising range of cognitive processes that can be explained by linkages to body systems, such as creativity, memory, and sensorimotor representations, which are essential for rebuilding and maintaining self-awareness of body consciousness.

These papers will stimulate interest in further basic and clinical research on the role of embodied cognition mechanisms and multisensory stimulation to support appropriate clinical treatment development regarding altered body representation, expanding our knowledge of this research topic.
